# Return to work after major depressive disorder: predictive factors and reintegration strategies

**DOI:** 10.1192/j.eurpsy.2026.11434

**Published:** 2026-07-31

**Authors:** C. Sridi, I. Kacem, N. Gannoun, R. Nakhli, A. Fki, F. Chelly, N. Belhadj, M. Maoua

**Affiliations:** 1 Occupational Medicine Department, Sahloul University Hospital; 2 University of Sousse, Faculty of Medicine of Sousse; 3 Research Laboratory LR19SP03; 4 Occupational Medicine Department, Farhat Hached University Hospital, Sousse, Tunisia

## Abstract

**Introduction:**

Major depressive disorder (MDD) is a leading cause of work disability and prolonged sickness absence worldwide. Sustainable return to work (RTW) after MDD is complex and influenced by clinical, personal, workplace and system-level factors. Occupational physicians play a central role in predicting RTW trajectories and coordinating reintegration strategies.

**Objectives:**

This review aims to synthesize current evidence on predictors of RTW following MDD and to identify effective reintegration strategies that can guide occupational health practice.

**Methods:**

We performed a narrative literature review of studies published between 2000 and 2024 using PubMed, PsycINFO and Web of Science. Search terms included “major depressive disorder”, “return to work”, “predictors”, “reintegration”, “occupational therapy”, and “vocational rehabilitation.” We prioritized systematic reviews, longitudinal cohort studies and randomized trials addressing predictors of RTW and effectiveness of RTW interventions.

**Results:**

Key predictors associated with lower probability or delayed RTW included greater baseline symptom severity, higher functional impairment, prior/longer sickness absence, older age, and certain personality traits (e.g., neuroticism). By contrast, positive RTW expectations, higher general and RTW-specific self-efficacy, higher workability scores and conscientiousness consistently predicted earlier RTW. Workplace factors, supportive supervision, graded re-entry, and reasonable accommodations, also improved outcomes.

Intervention evidence indicates that combined clinical (psychotherapy/medication) plus work-directed approaches (stakeholder dialogue, graded exposure, occupational therapy/coordination) reduce days of sickness absence and improve work functioning more effectively than clinical care alone. Purely work-directed interventions show mixed results unless integrated with clinical treatment and workplace engagement. Structured RTW programs, case management and occupational therapy have shown promising effects on sustainable RTW.

**Conclusions:**

RTW after MDD depends on clinical, individual, and workplace factors. Occupational physicians should promote integrated approaches combining treatment, workplace engagement, and graded reintegration to support sustainable return. Developing validated RTW-prediction tools and standardized reintegration models remains a priority.

**Disclosure of Interest:**

None Declared

